# ENO1 Binds to ApoC3 and Impairs the Proliferation of T Cells via IL-8/STAT3 Pathway in OSCC

**DOI:** 10.3390/ijms232112777

**Published:** 2022-10-24

**Authors:** Jing Wang, Qiwen Man, Niannian Zhong, Hanqi Wang, Chenxi Zhang, Suran Li, Linlin Bu, Bing Liu

**Affiliations:** 1 The State Key Laboratory Breeding Base of Basic Science of Stomatology (Hubei-MOST) & Key Laboratory of Oral Biomedicine Ministry of Education, School & Hospital of Stomatology, Wuhan University, Wuhan 430079, China; 2 Department of Oral and Maxillofacial Head Neck Oncology, School & Hospital of Stomatology, Wuhan University, Wuhan 430079, China

**Keywords:** oral squamous cell carcinoma, postoperative lymphatic drainage, ENO1, ApoC3

## Abstract

Lymph node metastasis is associated with poor prognosis of oral squamous cell carcinoma (OSCC), and few studies have explored the relevance of postoperative lymphatic drainage (PLD) in metastatic OSCC. Alpha-enolase (ENO1) is a metabolic enzyme, which is related to lymphatic metastasis of OSCC. However, the role of ENO1 in PLD in metastatic OSCC has not been elucidated. Herein, we collected lymphatic drainage after lymphadenectomy between metastatic and non-metastatic lymph nodes in OSCC patients to investigate the relationship between ENO1 expression and metastasis, and to identify the proteins which interacted with ENO1 in PLD of patients with metastatic OSCC by MS/GST pulldown assay. Results revealed that the metabolic protein apolipoprotein C-III (ApoC3) was a novel partner of ENO1. The ENO1 bound to ApoC3 in OSCC cells and elicited the production of interleukin (IL)-8, as demonstrated through a cytokine antibody assay. We also studied the function of IL-8 on Jurkat T cells co-cultured with OSCC cells in vitro. Western blot analysis was applied to quantitate STAT3 (signal transducer and activator of transcription 3) and p-STAT3 levels. Mechanistically, OSCC cells activated the STAT3 signaling pathway on Jurkat T cells through IL-8 secretion, promoted apoptosis, and inhibited the proliferation of Jurkat T cells. Collectively, these findings illuminate the molecular mechanisms underlying the function of ENO1 in metastasis OSCC and provide new strategies for targeting ENO1 for OSCC treatment.

## 1. Introduction

Oral squamous cell carcinoma (OSCC) is one of the most common malignant neoplasia, resulting in 377,713 new cases of OSCC and 177,757 deaths worldwide in 2020 [1,2]. Although OSCC resembles the cutaneous squamous cell carcinoma (CSCC) in the biological behaviors as two representative non-melanoma skin cancers [3], such as the high risk of local invasion and metastasis, the risk factors of them are quite different. While OSCCs are mainly caused by alcohol abuse, tobacco use, genetic alterations, and human papilloma virus (HPV) infection, continuous exposure to sunlight is an essential factor associated with CSCC [4,5]. The high mortality rate of OSCC is mainly due to the recurrence and metastasis of tumors [6,7]. Cervical lymph nodes are the most common site of OSCC metastasis [8], which may induce tumor-immune tolerance and promote distant metastasis [9]. A large number of lymphatic capillaries are ruptured during the OSCC surgery, and this condition results in postoperative lymphatic drainage (PLD) overflow [10]. In the clinical setting, drainage obtained from the anatomical sites of the excised lymph nodes has been used for diagnosis of diseases, such as melanoma [11,12,13]. However, studies on the PLD of human OSCC are very few.

Alpha-enolase (ENO1) has been reported as a glycolytic enzyme inducing tumorigenesis and tumor invasion under pathological conditions and mediating metabolic reprogramming in cancer cells that performs crucial roles in aerobic glycolysis and acts as a key contributor to the “Warburg effect” in cancer cells [14,15,16,17,18]. Overexpression of ENO1 was related to lymph node metastasis of multifarious malignant neoplasms, such as breast cancer and pancreatic cancer [19,20]. The ENO1 was also highly expressed in metastatic head and neck cancer cells, and the roles of ENO1 in head and neck cancer and oral squamous cell carcinoma have also been reported in promoting the occurrence and enhancing the lymphatic invasion [21,22,23]. However, the downstream mechanism of ENO1 in the lymphatic metastasis in OSCC, especially the role in the PLD, has not been elucidated.

Apolipoprotein B (ApoB), which is a low-density lipoprotein component, could bind to ENO1 to promote the secretion of inflammatory factors and aggravate arthritis [24]. Apolipoprotein C-III (ApoC3), which is a component of a very low-density lipoprotein that plays a major role in lipoprotein metabolism, can activate NLRP3 to promote aseptic inflammation and organ damage [25]. However, the role of ApoC3 in OSCC is still unclear. The crosstalk between ENO1 and ApoC3 in human OSCC PLD has not been reported in detail. Inflammation can increase the risk of cancer by providing cells with cytokines that infiltrate the tumor microenvironment [26]. It has been increasingly recognized that tumor microenvironment plays an important role in carcinogenesis. During carcinogenesis, an inflammatory component is present and contributes to tumor proliferation, angiogenesis, metastasis, and resistance to hormonal and chemotherapy [27]. Interleukin (IL)-8 is a cytokine involved in the infectious responses and the pathogenesis of multiple inflammatory diseases and cancers [28]. It was reported to be associated with immunosuppression, and increased tumor IL-8 levels were negatively correlated with T cell infiltration-related transcription markers [29]. Therefore, the identification of this inflammatory mediator that induces inflammation in OSCC patients is crucial for the discovery of specific therapeutic targets. The signal transducer and activator of the transcription 3 (STAT3) signaling pathway also plays an important role in regulating the anti-tumor-immune response, and phosphorylated STAT3 (p-STAT3) could impair tumor–immune surveillance and promote tumor escape [30,31]. Currently, few studies on the relationship between IL-8 and STAT3 have been reported [32,33].

In this study, we evaluated ENO1 expression in OSCC, its relationship with the clinal stages of OSCC patients, and the expression of ENO1 in metastatic lymph nodes. We also collected PLD after lymphadenectomy between lymph nodes metastatic (LN+ group) and non-metastatic (LN− group) OSCC patients and found high ENO1 expression in the PLD of metastatic OSCC. Thus, we sought to identify the proteins which interacted with ENO1 in the PLD of patients with metastatic OSCC by MS/GST pulldown assay to explore the role ENO1 plays in PLD. Here, ApoC3 was identified as potent partner of ENO1 in PLD of patients with metastatic OSCC. In view of the fact that the interaction between ENO1 and ApoC3 remained unclear in the genesis and development of OSCC, we demonstrated in this study that ENO1 bound to ApoC3 in OSCC cells and elicited the production of IL-8, which activated the STAT3 signaling pathway in Jurkat T cells. It then promoted the apoptosis and inhibited the proliferation of Jurkat T cells. Collectively, these findings contribute to understanding the relationship between ENO1, which could be considered as a promising target, and the occurrence, development, and metastasis of OSCC.

## 2. Results

### 2.1. ENO1 Was Overexpressed in Human OSCC Tissues

**Figure 1:**
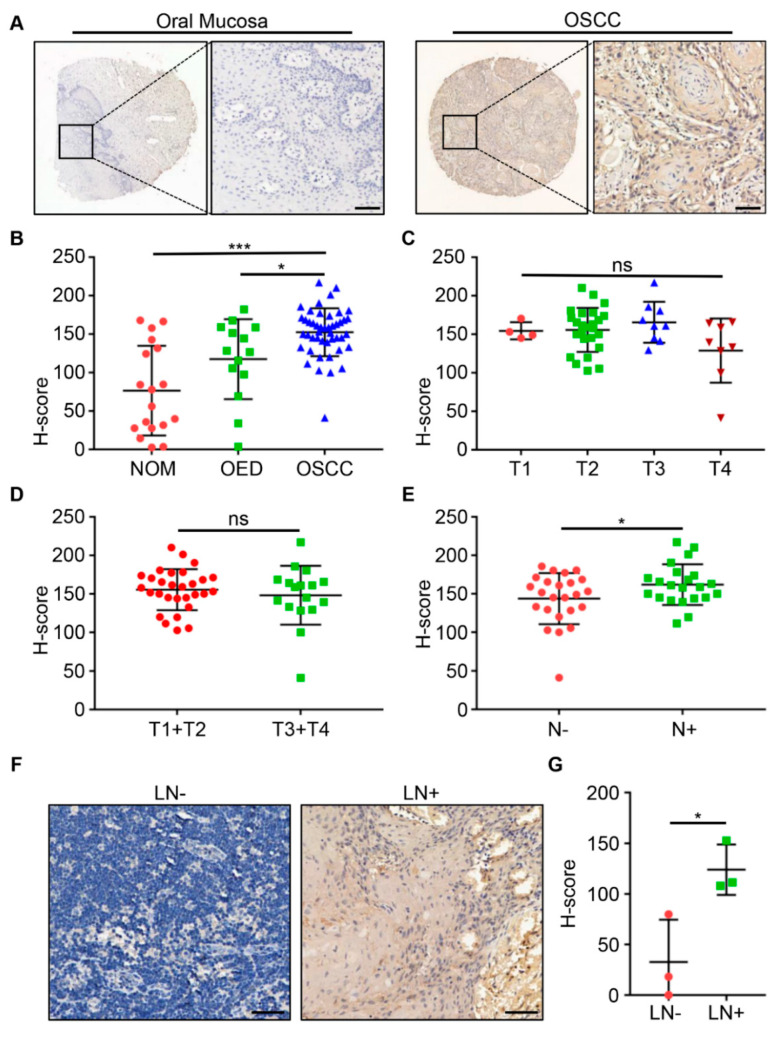
Overexpression and clinicopathological significance of ENO1 in primary oral squamous cell carcinoma (OSCC). (**A**) Representative immunohistochemical (IHC) staining of ENO1 in oral mucosal tissue and OSCC tissue. Scale bar, 50 µm. (**B**) Quantitative analysis of ENO1 expression in normal oral mucosa (NOM), oral epithelial dysplasia (OED), and OSCC tissues. (**C**) Quantitative analysis of ENO1 expression in OSCC classified by tumor sizes (T1, T2, T3, and T4). (**D**) Quantitative analysis of ENO1 expression in OSCC classified by tumor sizes (T1+T2, T3+T4). (**E**) Quantitative analysis of ENO1 expression in non-metastatic (left) and metastatic (right) OSCC tissues. (**F**) Representative IHC staining images of ENO1 in non-metastatic lymph node (LN−) and metastatic lymph node (LN+). Scale bar, 50 µm. (**G**) Quantitative analysis of ENO1 expression in non-metastatic and metastatic lymph nodes. * *p* < 0.05, *** *p* < 0.001; ns is not significant.

The IHC staining was performed on tissue microarrays, which were characterized by the cytoplasmic expression, to detect the ENO1 expression level in human OSCC and normal oral mucosa (NOM) (Figure 1A). Notably, the staining intensity of ENO1 in OSCC was discovered to be rather stronger than that in NOM (*p* < 0.001) and oral epithelial dysplasia (OED) (*p* < 0.05, Figure 1B). Western blot analysis also showed that ENO1 was highly expressed in OSCC cell lines (Figure S1A).

### 2.2. Clinicopathological Correlation of ENO1 Expression Level in OSCC

**Figure 2:**
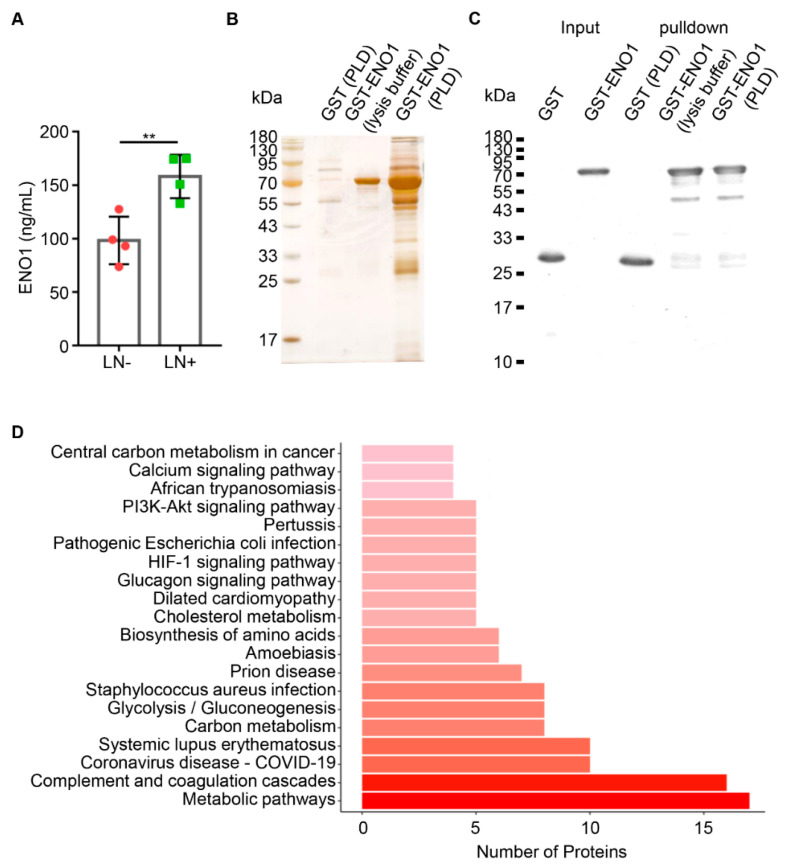
ENO1 directly bound to ApoC3 in postoperative lymphatic drainage (PLD) of metastatic OSCC. (**A**) Quantitative analysis of ENO1 expression in PLD between non-metastatic (left) and metastatic (right) OSCC patients. (**B**) The SDS-PAGE for the GST pulldown product shown by silver staining. (**C**) Western blot analysis using an anti-GST antibody for the GST pulldown product. (**D**) Distribution of the top 20 KEGG pathways based on ENO1-interacting proteins in PLD of metastatic OSCC. Columns refer to related pathways, which are colored with gradient colors from midnight red (metabolic pathways) to lighter red (central carbon metabolism in cancer). ** *p* < 0.01.

The relationship between the expression of ENO1 and pathological features was studied, and no obvious correlation between ENO1 and the OSCC T stages (T1 vs. T2 vs. T3 vs. T4; T1+T2 vs. T3+T4; *p* > 0.05, Figure 1C,D) was found. Interestingly, ENO1 expression was higher in patients with lymph node metastasis than in those without (*p* < 0.05, Figure 1E) and the expression level of ENO1 was higher in the primary lesions of OSCC patients with pathologically positive lymph node metastasis by IHC staining (Figure S1B). Furthermore, a stronger IHC staining of ENO1 was observed in metastatic lymph nodes than in non-metastatic lymph nodes (*p* < 0.05, Figure 1F,G), indicating a positive correlation between the higher ENO1 expression and the metastatic lymph node status. In addition, the ELISA results revealed that the ENO1 level in PLD from metastatic OSCC patients was higher than that from non-metastatic OSCC patients (*p* < 0.01, Figure 2A).

### 2.3. ENO1 Directly Interacted with ApoC3 in OSCC

Proteins from the PLD of patients with metastatic OSCC were isolated by pulldown assay using the recombinant protein GST-ENO1 as an acceptor to incubate the PLD for identifying proteins interacting with ENO1 (Figure 2B,C). Potential proteins were precipitated with the GST-ENO1 and identified by mass spectrometry (MS). Of the ENO1 binding proteins, 100 proteins were significantly enriched in OSCC PLD (Table S1). Among them, 10 proteins with relatively higher coverage of the polypeptide and peptide sequences with ENO1 are shown in Table 1. The KEGG analysis revealed the top 20 significant pathways based on ENO1-interacting proteins in the PLD of metastatic OSCC (Figure 2D). Metabolic pathways were the most significantly enriched pathways. The complete list of the enriched KEGG pathways of ENO1 interacting proteins was presented in Table S2. A protein–protein interaction (PPI) network associated with ENO1 proteins was also generated and examined using STRING database (version 11.5, https://string-db.org/), as presented in Figure S2.

Here, ApoC3, which is crucial in the development of inflammation, was screened as a potential metabolism-related partner protein that showed high coverage with ENO1 (Table 1). Then, the ENO1 and ApoC3 interaction was examined using double-labelling immunofluorescence on the human OSCC cell line CAL27 and the OSCC tissue (Figure S3A,B), showing that ApoC3 and ENO1 were co-localized in the CAL27 cells and the OSCC tissue.

### 2.4. ApoC3 was Overexpressed in Human OSCC

**Figure 3:**
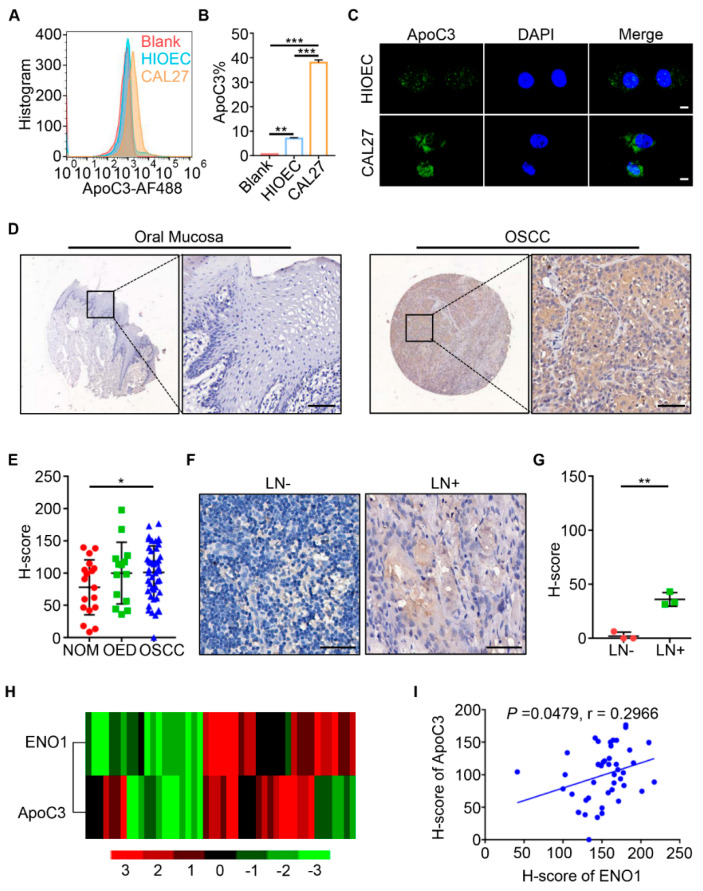
Overexpression and clinicopathological significance of ApoC3 in OSCC. (**A**,**B**) Flow cytometry analysis of the expression of ApoC3 in HIOEC and CAL27 cells. (**C**) Fluorescence confocal microscopy of the expression of ApoC3 in HIOEC and CAL27 cells. Scale bar, 10 µm. (**D**) Representative IHC staining of ApoC3 in oral mucosal tissue and OSCC tissue. Scale bar, 50 µm. (**E**) Quantitative analysis of ApoC3 expression in NOM, OED, and OSCC. (**F**) Representative IHC staining of ApoC3 in non-metastatic lymph node (LN−) and metastatic lymph node (LN+). Scale bar, 50 µm. (**G**) Quantitative analysis of ApoC3 expression in non-metastatic and metastatic lymph nodes. (**H**) Hierarchical clustering showing the correlation between ENO1 and ApoC3 in OSCC. (**I**) Correlation coefficient test of ENO1 with ApoC3 evaluated by Pearson’s analysis (*p* = 0.0479, r = 0.2966). * *p* < 0.05, ** *p* < 0.01, *** *p* < 0.001.

Flow cytometry was used to analyze the membrane ApoC3 expression in the OSCC cell line CAL27 and the human immortalized oral epithelial cell line HIOEC. The results showed that the ApoC3 expression was higher in CAL27 cells (Figure 3A,B, *p* < 0.001). Under fluorescence confocal microscopy, ApoC3 was highly expressed in the cell membrane of CAL27 cells, but rarely observed in HIOEC cells (Figure 3C). Figure S4A showed the same trend of the ApoC3 expression in CAL27 cells and HIOEC cells under fluorescence microscopy. The ApoC3 expression was examined using IHC staining on a tissue microarray, and the results showed that the ApoC3 expression in OSCC tumor cells was much stronger than that in NOM (*p* < 0.05, Figure 3D,E). Furthermore, ApoC3 expression was significantly higher in metastatic lymph nodes than in non-metastatic (*p* < 0.01, Figure 3F,G), which indicated the positive correlation between the ApoC3 expression and the lymph node status. However, no significant difference was observed in the correlations between the ApoC3 expression and the T stages (T1 vs. T2 vs. T3 vs. T4; T1+T2 vs. T3+T4; *p* > 0.05, Figure S4B,C) of OSCC. Hierarchical clustering analysis showed a similar trend between ENO1 and ApoC3 expression (Figure 3H), and the statistical correlation between them was demonstrated using Pearson’s correlation analysis (*p* = 0.0479, r = 0.2966, Figure 3I).

### 2.5. ENO1 Binding to ApoC3 Mediated the Production of Proinflammatory Cytokines

**Figure 4:**
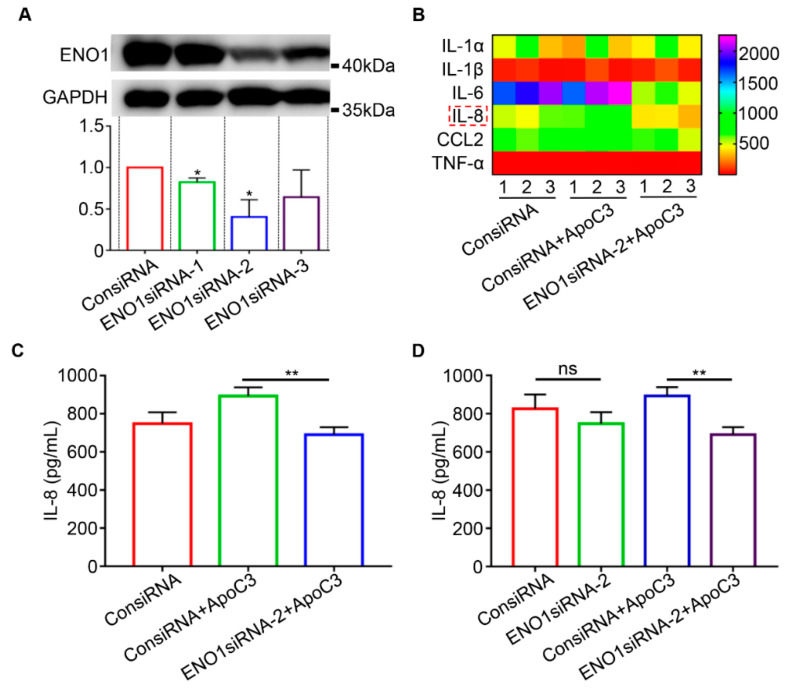
ENO1 induced proinflammatory cytokines in CAL27 supernatant and was dependent on ApoC3. (**A**) Western blot analysis for verifying ENO1 expression after control siRNA (ConsiRNA) and ENO1siRNA-1/2/3 transfection in CAL27 cells. (**B**) Heatmap of the production levels of the inflammatory cytokines in the cultured CAL27 cell supernatant, tested through a QAH-INF-1 cytokine antibody array. (**C**) Quantification of IL-8 production levels in the cultured CAL27 cell supernatant determined by the inflammatory cytokine assay. (**D**) Quantification of IL-8 production levels in the cultured CAL27 cell supernatant determined by ELISA. ** *p* < 0.01; ns is not significant. Each experiment was repeated at least three times.

Three siRNAs were designed targeting ENO1 (ENO1siRNA-1/2/3) knockdown, and their working efficiencies were examined in CAL27 cells using Western blot analysis (Figure 4A). Here, ENO1siRNA-2 worked the best and was, thus, used for subsequent experiments. The effects of ENO1 binding to ApoC3 on inflammatory cytokines production in cultured CAL27 cells was tested through a QAH-INF-1 cytokine antibody array. The production results of IL-1α, IL-1β, IL-6, IL-8, CCL2, and TNF-α were presented as a heatmap and quantified, as shown in Figure 4B and Figure S5A–F, respectively. It was showed that increased IL-8 was produced in CAL27 cells in response to the ApoC3 stimulation; in the meantime, the production of IL-8 in CAL27 cells was suppressed when ENO1 was downregulated (Figure 4C). In addition, it was demonstrated by the ELISA result that the IL-8 production was suppressed under the circumstance of ENO1siRNA-2 treatment (Figure 4D), in accordance with the result of the cytokine antibody array. The ELISA result showed that the IL-8 production levels were not significantly different between the control group (ConsiRNA) and ENO1 knockdown group (ENO1siRNA-2); however, after being stimulated with human recombinant ApoC3 protein, the difference between these two groups was significant (ConsiRNA+ApoC3 vs. ENO1siRNA-2+ApoC3) and the IL-8 production showed a marked decrease (*p* < 0.01).

### 2.6. IL-8 Impaired the Proliferation of T Cells through the STAT3 Signaling Pathway

**Figure 5:**
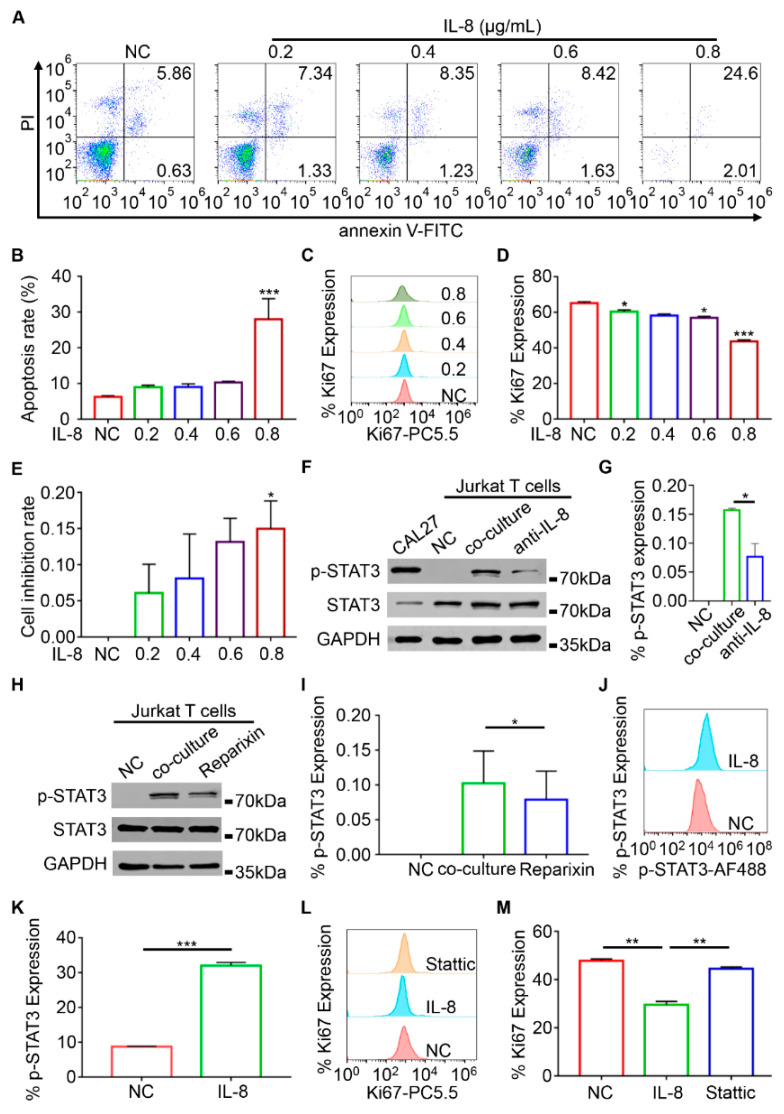
IL-8 inhibited the proliferation of T cells through the STAT3 signaling pathway. (**A**,**B)** Flow cytometry analysis for cell apoptosis of Jurkat T cells stimulated with human recombinant IL-8 proteins (0, 0.2, 0.4, 0.6, and 0.8 μg/mL). (**C**,**D**) Flow cytometry analysis for the expression of Ki67 in Jurkat T cells stimulated with human recombinant IL-8 proteins (0, 0.2, 0.4, 0.6, and 0.8 μg/mL). (**E**) Inhibition rate of Jurkat T cells stimulated with human recombinant IL-8 proteins (0, 0.2, 0.4, 0.6, and 0.8 μg/mL) determined by CCK-8 assay. (**F**,**G**) Western blot analysis for p-STAT3 levels in CAL27 cells without treatment, Jurkat T cells without treatment, Jurkat T cells co-cultured with CAL27 supernatant, and Jurkat T cells co-cultured with CAL27 supernatant and 0.5 μg/mL anti-IL-8 neutralizing antibody treatment. (**H**,**I**) Western blot analysis for p-STAT3 levels in Jurkat T cells without treatment, Jurkat T cells co-cultured with CAL27 supernatant, and Jurkat T cells co-cultured with CAL27 supernatant and 100 ng/mL reparixin (inhibitor of the IL-8 receptors CXCR1/2). (**J**,**K**) Flow cytometry analysis for p-STAT3 expression levels in Jurkat T cells without treatment and Jurkat T cells stimulated with 0.8 μg/mL recombinant human IL-8 proteins. (**L**,**M**) Flow cytometry analysis for p-STAT3 expression levels in Jurkat T cells without treatment, Jurkat T cells stimulated with 0.8 μg/mL recombinant human IL-8 proteins, and Jurkat T cells stimulated with 0.8 μg/mL recombinant human IL-8 proteins and 6 μmol/L stattic (inhibitor of STAT3). * *p* < 0.05, ** *p* < 0.01, *** *p* < 0.001. Here, NC is negative control.

The apoptosis rates of Jurkat T cells treated with various concentrations of recombinant human IL-8 proteins were analyzed by flow cytometry. The result showed that the apoptosis rate of Jurkat T cells increased with the rise in IL-8 concentration (Figure 5A). Remarkably, the apoptosis rate of Jurkat T cells was the highest when IL-8 was 0.8 μg/mL (*p* < 0.001, Figure 5B). The Ki67 expression levels of Jurkat T cells were also detected after the stimulation of different concentrations of IL-8, showing an inhibitory effect of IL-8 on Jurkat T cells, predominant at 0.8 μg/mL (*p* < 0.001, Figure 5C,D). Furthermore, the CCK-8 assay results suggested that the cell inhibition rate was gradually augmented with the increase in IL-8 concentration (Figure 5E). These results indicated that IL-8 has a dose-dependent inhibition effect on the proliferation of Jurkat T cells.

To investigate the underlying mechanism of IL-8 regulation on Jurkat T cells, *p*-STAT3 expression was detected using Western blot analysis and flow cytometry. The p-STAT3 expression level in Jurkat T cells was increased through the co-culture with CAL27 cells, but then decreased after the anti-IL-8 neutralizing antibody treatment (Figure 5F,G). Furthermore, reparixin, an inhibitor of the IL-8 receptors CXCR1/2, reduced p-STAT3 expression levels to varying degrees (*p* < 0.05, Figure 5H,I). In addition, the level of p-STAT3 was increased in Jurkat T cells after being stimulated with the recombinant human IL-8 protein (*p* < 0.001, Figure 5J,K). In the end, as shown in Figure 5L-M, the Ki67 expression level was restored by stattic, an inhibitor of STAT3, after a drop caused by the IL-8 treatment (*p* < 0.01, Figure 5L,M). Taken together, STAT3 played an important role in the process of IL-8 regulation on Jurkat T cells. Furthermore, IL-8 promoted the phosphorylation of STAT3 and impaired the proliferation of Jurkat T cells.

## 3. Discussion

Recurrence and metastasis are generally considered to be the major causes of OSCC-related deaths [7]. Cervical lymphatic metastasis is the most common form of metastasis, which is associated with poor prognosis [34,35]. Nowadays, surgery is still the main treatment for OSCC [36] and neck lymphadenectomy is an effective way to treat or prevent neck metastasis of OSCC [37]. In OSCC surgery, a large amount of capillary lymphatic vessels rupture, which causes PLD to be exuded from the surgical site [10]. The ENO1 can be expressed in the cytoplasm and cell membrane, and its overexpression is closely related to various tumors, such as lung cancer, oral cancer, and gastric cancer [38,39,40,41]. Indeed, ENO1, as a glycolytic enzyme, is a multifunctional oncoprotein, and its glycolytic function deregulates cellular energetic, sustains tumor proliferation, and is correlated with poor prognosis [39]. A previous study showed that ENO1 was overexpressed in head and neck cancer cells, highly expressed in patients with cervical lymphatic metastasis, and closely related to poor clinical outcomes [21].

In our study, a high expression of ENO1 in OSCC tissue was discovered. A pulldown/MS analysis was performed to identify the partner proteins of ENO1 in order to study its role in PLD in metastatic OSCC (Table 1). The results showed that ApoC3 had a high coverage rate with ENO1. Furthermore, ApoC3, which has an essential role in triglyceride-rich lipoprotein metabolism, promoted sterile inflammation and organ damage. The ApoC3 is expressed in hepatocytes and less in enterocytes [42]; it promotes the release of ApoC3-induced IL-1β in human monocytes with TLR2 and TLR4 acting as its receptors [43]. In this study, the immunofluorescence staining and cytokine antibody array showed that ENO1 bound to ApoC3 in OSCC cells and, thereafter, increased the release of IL-8. Proinflammatory cytokines are important regulators of cancer metastasis [44,45]. IHC analysis showed a significantly higher ApoC3 expression in human OSCC tissues than in NOM. The upregulation of ApoC3 in cancer tissues has been reported in cancers including hepatocellular carcinoma [46] and bladder cancer [47]; besides, a significant decline of ApoC3 associated to colorectal cancer progression was also reported, which provides a significant statistical value for diagnosis [48]. To the best of our knowledge, this study is the first to explore ApoC3 expression in human OSCC and to evaluate the clinicopathological value of ApoC3.

Furthermore, IL-8, which is produced by monocytes, endothelial cells, and various epithelial cells, is involved in infectious responses and the pathogenesis of various inflammatory diseases and cancers [28]. Studies revealed that IL-8 could promote the progression of multiple human cancers including prostate cancer [49], non-small cell lung carcinoma [50], gastric cancer [51], thyroid cancer [52], and OSCC [53,54]. In cancers, IL-8 production is primarily dependent on tumor cells, and a correlation between its concentration with tumor progression has been indicated. Tumor-derived IL-8 can shape the immune microenvironment to enhance the invasive ability and induce angiogenesis [55]. Inflammation-associated colonic and gastric carcinogenesis was promoted through IL-8 increasing the recruitment of myeloid-derived suppressor cells throughout the inflammatory response [56]. Moreover, a close correlation was observed between elevated serum IL-8 and bulk tumor IL-8 gene expression, as well as decreased intratumoral T cells, which suggests the immunosuppression function of IL-8 [57]. However, our study still has some limitations, such as lack of the prognostic information owing to short follow-up time and the small number of patients included. Moreover, we could not confirm whether the in vivo effects of ENO1 and ApoC3 agree to the in vitro effects; a further study on the role of ENO1 and ApoC3 in experimental animals is needed.

The Janus kinase (JAK)-mediated STAT3 tyrosine phosphorylation signaling cascade is an important pathway triggered by IL-8, as previously reported in esophageal squamous cell carcinoma and prostate tumor [33,58]. Here, we demonstrated through Western blot analysis or flow cytometry that the level of p-STAT3 in Jurkat T cells increased after IL-8 stimulation. Furthermore, the addition of IL-8 neutralizing antibodies or reparixin (the inhibitor of the IL-8 receptor “CXCR1/2”) restored the p-STAT3 level. Further studies revealed that stattic, a STAT3 inhibitor could also restore the expression of Ki67. Whether STAT3 acts by binding directly to Ki67 or by some other mechanism requires further study. These data suggest that IL-8 promote STAT3 phosphorylation to impair Jurkat T cells activity and proliferation, indicating a new pathway of tumor immune escape during OSCC progression.

In conclusion, this study showed high ENO1 expression in the PLD of metastatic OSCC and demonstrated that ENO1 bound to ApoC3 in PLD. This combination promoted IL-8 secretion in OSCC cells. We studied IL-8 function in Jurkat T cells through co-culture in vitro with OSCC cells (CAL27 cell line) and its relationship with the downstream STAT3/p-STAT3 pathway. The results showed that OSCC cells activated the STAT3 signaling pathway on Jurkat T cells through IL-8 secretion, which promoted the apoptosis and inhibited the proliferation of Jurkat T cells. These findings illuminate the molecular mechanisms underlying the function of ENO1 in metastasis OSCC and provide new strategies for targeting ENO1 for OSCC treatment.

## 4. Materials and Methods

### 4.1. Patient and Sample Collection

The metastatic lymph node (LN+) and non-metastatic lymph node (LN−) were collected from patients with OSCC, fixed with 4% paraformaldehyde and paraffinized. Four samples of lymphatic drainage were collected after lymphadenectomy of OSCC patients. The procedures were implemented in accordance with the National Institutes of Health guidelines regarding the use of human tissues. This study was approved by the review board of the Ethics Committee of the Hospital of Stomatology, Wuhan University (2018LUNSHENZI#A24, approved on February 28, 2018). All subjects provided informed consent in our research.

### 4.2. Human OSCC Tissue Microarrays

Specimens were collected from OSCC patients in the Department of Oral and Maxillofacial Head Neck Oncology, School and Hospital of Stomatology, Wuhan University. Specimens in the tissue microarrays comprised OSCC primary lesions, normal oral mucosa (NOM) adjacent, and oral epithelial dysplasia (OED) lesion. The OSCC stages were determined according to the TNM staging system developed by the guidelines and the UICC pathological classification systems [59].

### 4.3. Immunohistochemical (IHC) Staining

The method of IHC staining was conducted as described previously [60]. The tissue microarrays were primarily incubated with ApoC3 antibodies (rabbit; #GTX129994; GeneTex, Irvine, CA, USA) and ENO1 (mouse; #MAB11222; Abnova, Taipei, Taiwan), respectively, at 4 °C overnight. The Aperio ScanScope CS scanner (Sausalito, CA, USA) was used for slice scanning, and the Aperio Quantification software was used for slice quantification. The histoscore (H-score) was obtained by applying the following formula [61]: H-score = [(1+) × 1 + (2+) × 2 + (3+) × 3]. Cluster 3.0 with average linkage was used for hierarchical clustering analysis, and data were obtained using Java TreeView 1.1.3 (developed by Alok J. Saldanha, Stanford University, Stanford, CA, USA).

### 4.4. Human Lymphatic Drainage

A volume of 5–10 mL of postoperative lymphatic drainage was collected from 24–48 h after lymphadenectomy. Using the Centrifuge 5810R (Eppendorf, Hamburg, Germany), samples were centrifuged at 500× *g* for 10 min at 4 °C and the supernatant fractions were centrifuged at 3000× *g* for another 20 min to remove large cell debris. Thereafter, 3 mL of cell-free lymphatic drainage was stored at −80 °C for subsequent experiments.

### 4.5. GST Pulldown Assay

The GST-ENO1 and GST were respectively inserted into vector pGEX-4T-1 (FitGene Biotechnology Co., Ltd., Guangzhou, China), which were transformed into *Escherichia coli* (*E. coli*) BL21 (FitGene Biotechnology Co., Ltd., Guangzhou, China), and the recombinant ENO1 proteins were expressed as GST fusions. Then, *E. coli* BL21 was cultured overnight in Luria–Bertani broth with ampicillin at 37 °C. In this study, 0.1 mM of isopropyl-β-d-thiogalactoside was used for recombinant protein induction for 3 h at 20 °C. Furthermore, *E. coli* BL21 was lysed using lysis buffer (FitGene Biotechnology Co., Ltd., Guangzhou, China) and ultrasonic cracking. A total of 50 μL of the lysate supernatant was taken as “input”. Then, GST-resins (GE Healthcare, Waukesha, WI, USA) were washed by lysis buffer twice and incubated with the lysate supernatant at 4 °C for 2 h, and they were used as GST (PLD), GST-ENO1 (lysis buffer), and GST-ENO1 (PLD). Subsequently, the resins were washed twice and then incubated with the lymphatic drainage samples at 4 °C overnight. Finally, the GST-labelled resins were washed by 20 mM reduced glutathione (Sigma, Darmstadt, Germany) for 10 min. The pulldown products were collected and stored at −80 °C. The proteins were analyzed using western blot analysis targeting GST antibody (mouse; #HT601; TransGen Biotech, Beijing, China) and SDS-PAGE and silver staining.

### 4.6. Mass Spectrometry

The LC–MS/MS analysis of the tryptic peptides was conducted on a Dionex Ultimate 3000 RSLCnano system (Thermo Scientific, Waltham, MA, USA) coupled with a Q Exactive mass spectrometer (Thermo Scientific, Waltham, MA, USA). The peptides were eluted from the Acclaim PepMap RSLC C18 nanoscale analytical column (Thermo Scientific, Waltham, MA, USA) with a gradient from 5% to 90% acetonitrile over 65 min at a flow rate of 300 nL/min. The primary parameters were set as follows: AGC target of 3e6, maximum IT of 40 ms, and a full MS scan range of 350–1800 m/z with a resolution of 70,000. The secondary parameters were set as follows: AGC target of 1e5, maximum IT of 60 ms, TopN of 20, NCE/stepped NCE of 27, and a resolution of 17,500.

The acquired raw spectral data were processed with the Proteome Discoverer 1.4 software (Version 1.4.0.288, Thermo Fisher, Waltham, MA, USA) and ProteinPilot™ Software 4.5 (Version 1656, AB Sciex, Framingham, MA, USA) for MS. The database used for retrieval and sequence alignment was as follows: PR1-21030019-uniprot-taxonomy__Homo_sapiens-reviewed_200601.fasta.

### 4.7. KEGG Pathway Enrichment and PPI Network Analysis

Kyoto Encyclopedia of Genes and Genomes (KEGG; www.genome.jp/kegg/pathway.html, accessed on 27 January 2021) pathway enrichment analysis was conducted using KOBAS 3.0 [62,63,64,65], and the protein–protein interaction (PPI) network of ENO1 interacting proteins was generated by the STRING database (http://string-db.org/, accessed on 30 August 2022) [66] and analyzed with STRING database (version 11.5) [67].

### 4.8. Cell Culture

The human OSCC cell line CAL27 (obtained from the China Center for Type Culture Collection) and the human acute T lymphocytic leukemia cell line (Jurkat T cells) (obtained from American Type Culture Collection, Rockville, MA, USA) were grown in DMEM (Gibco, Waltham, MA, USA) and RPMI 1640 medium (Gibco, Waltham, MA, USA), respectively. The two kinds of medium were both augmented with 10% fetal bovine serum (FBS) as a supplement (CellMax, Shenzhen, China). Human immortalized oral epithelial cell line (HIOEC) was provided by Professor Zhi-Jun Sun (School and Hospital of Stomatology, Wuhan University, Wuhan, China) and was cultured in KGM™ Gold (Lonza, Walkersville, MD, USA). All cells were incubated at a 37 °C humidified atmosphere containing 5% CO_2_.

### 4.9. Immunofluorescence

Double-labelling immunofluorescence staining procedures on CAL27 cells and paraffin-embedded tissue samples were conducted as described previously [60,68]. The cells and the slides were incubated with the primary antibodies against ENO1 and ApoC3, respectively, at 4 °C overnight. Immunofluorescence staining procedures on HIOEC and CAL27 cells were also performed, as described previously [69]. The HIOEC and CAL27 cells were incubated primarily with the ApoC3 antibody at 4 °C overnight. The secondary antibodies (goat anti-rabbit-IgG-Alexa Fluor 488; #A23220; goat anti-mouse-IgG-Alexa Fluor 594; #A23410; Abbkine, Wuhan, China) were incubated at 37 ℃ for 20 min, respectively. Cell nuclei were stained by 4′,6-diamidino-2-phenylindole (DAPI). Images were visualized using fluorescence microscopes.

### 4.10. Confocal Microscopy

Laser confocal dishes of 20 mm (NEST Biotechnology, Beijing, China) were used for the culture of HIOEC and CAL27 cells. Permeation of 15 min at RT of 4% paraformaldehyde in phosphate buffered saline (PBS) was used. The primary ApoC3 antibody was incubated at 4 °C overnight. Fluorescence-labeled secondary antibodies were then incubated at 37 °C for 20 min. Cell nuclei were stained by DAPI. A confocal microscope (Eclipse C2, Nikon, Tokyo, Japan) was used to capture images.

### 4.11. Flow Cytometry

After trypsin digestion, the collected single-cell suspensions of HIOEC and CAL27 cells were incubated with ApoC3 antibody at 4 °C for 1 h, followed by the corresponding fluorescein-conjugated secondary antibody (goat anti-rabbit-IgG-Alexa Fluor 488). In the end, cell suspensions were detected and analyzed using the flow cytometer (CytoFLEX, Beckman Coulter, CA, USA) and FlowJo 10 (VX.0.7, Tree Star, Ashland, OR, USA), respectively. Each procedure was followed by PBS washing, which was carried out three times.

Jurkat T cells were seeded in 6-well culture plates and stimulated by a graded concentration of recombinant human IL-8 (0.2, 0.4, 0.6, and 0.8 μg/mL) (#200-08; PeproTech, London, UK). Harvested and washed cells were suspended with 100 μL of binding buffer as per the instruction of the annexin V-FITC/PI cell apoptosis detection kit (4A Biotech, Beijing, China), Then, they were incubated with 5 μL of annexin V/FITC staining solution and 10 μL of propidium iodide (PI) buffer successively, which was conducted away from light. The early and late apoptotic cells were detected and analyzed using the flow cytometer and FlowJo 10 software. In the same way, Jurkat T cells were stained with the PC5.5-conjugated Ki67 antibody (#350519, BioLegend, San Diego, CA, USA), both at 4 °C for 1h and then they were run on the flow cytometer and analyzed.

Jurkat T cells were seeded in 6-well culture plates and stimulated by recombinant human IL-8 (0.8 μg/mL), The collected single-cell suspensions were suspended in 100 μL of PBS. An appropriate amount of p-STAT3 antibody (rabbit; #9145; Cell Signaling Technology, Danvers, MA, USA) was added and incubated at 4 °C for 1 h, followed by the corresponding fluorescein-conjugated secondary antibody (goat anti-rabbit-IgG-Alexa Fluor 488) for 1 h. Then, they were run on the flow cytometer and analyzed using FlowJo 10 (VX.0.7) software (Tree Star, Ashland, OR, USA).

Jurkat T cells were seeded in 6-well culture plates and stimulated with recombinant human IL-8 (0.8 μg/mL), followed by stattic (#A2224; APExBIO; Houston, TX, USA). The collected single-cell suspensions were stained with the PC5.5-conjugated Ki67 antibody (#350519, BioLegend, San Diego, CA, USA) at 4 °C for 1h, before they were run on the flow cytometer and analyzed using FlowJo 10 (VX.0.7) software.

### 4.12. Co-Culture of Jurkat T and CAL27 Cells

Jurkat T cells were cultured with the CAL27 supernatant for 2 h. According to the group, IL-8 neutralizing antibody (0.5 μg/mL) (#GTX10768; GeneTex, Irvine, CA, USA) or reparixin (100 ng/mL) (#A3752; APExBIO; Houston, TX, USA) was added or not.

### 4.13. SiRNA Knockdown of ENO1

The CAL27 cells were grown to 50% confluence before the transfection of the small interfering RNAs (siRNAs) for silencing ENO1 (Ribobio Co., Ltd., Guangzhou, China). Four groups were classified, as follows: NC (negative control oligonucleotides), ENO1siRNA-1 (target sequence—AGTCCTTCATCAAGGACTA), ENO1siRNA-2 (target sequence—GCTGCTGAAGACTGCTATT), and ENO1siRNA-3 (target sequence—GGAAGTATGACCTGGACTT). These groups were transfected at a final concentration of 50 nM into CAL27 cells at 37 °C for 24 h. The Ribo FECT^TM^ CP Transfection Kit (RiboBio Co., Ltd., Guangzhou, China) was used.

### 4.14. Western Blot Analysis

Western blot analysis was performed as described previously [61]. The primary antibodies used were as follows: ENO1 antibody (mouse; #MAB11222; Abnova, Taipei, Taiwan), STAT3 antibody (mouse; #9139; Cell Signaling Technology), p-STAT3 antibody (rabbit; #9145; Cell Signaling Technology, Danvers, MA, USA), and GAPDH antibody (rabbit; #GB11002; Servicebio, Wuhan, China) at 4 °C overnight. The membranes were visualized using the ECL detection system (SigmaAldrich, Darmstadt, Germany), as described previously (Man et al., 2019).

### 4.15. Inflammatory Cytokine Assay

The CAL27 cells were transfected with ENO1siRNA-2 or control siRNA (ConsiRNA) 24 h and were then cultured in an FBS-free DMEM with human ApoC3 recombinant protein (5 μg/mL) (#H00000345-Q01, Abnova, Taipei, Taiwan) at 37 °C for 24 h. The culture supernatants were collected and placed at −80 °C for the analysis of inflammatory cytokines. This was performed using the QAH-INF-1 array (Raybiotech, Peachtree Corners, GA, USA) following the instruction of the manufacturer (Wayen Biotechnologies Inc., Shanghai, China).

### 4.16. Enzyme-Linked Immunosorbent Assay (ELISA)

The levels of ENO1 in the PLD of metastatic and non-metastatic OSCC patients were quantified by an alpha-enolase (ENO1) ELISA kit (CSB-E17177h; Cusabio, Houston, TX, USA). The dilution ratio of the PLD samples was 1:20.

Similarly, a human-specific IL-8 ELISA kit (Beijing 4A Biotech Co., Ltd., Beijing, China) was used to determine the amount of IL-8 inflammatory cytokine in the culture supernatant produced by the CAL27 cells, which were transfected with ENO1siRNA-2 or ConsiRNA for 24 h and then cultured in FBS-free DMEM with human ApoC3 recombinant protein (5 μg/mL) at 37 °C for 24 h.

### 4.17. Cell Cytotoxicity Assay

Jurkat T cells were stimulated with different concentrations of recombinant human IL-8 (0.2, 0.4, 0.6, 0.8 μg/mL) for 24 h in a 96-well culture plate. A total of 10 μL of CCK-8 solution (Cell Counting Kit-8, Dojindo, Japan) was added to each well, before the resultant was cultured at 37 °C for 4 h. The absorbance value of 450 nm was determined with the Thermo MutliscanMK3 micro-plate reader (Thermo Fisher Scientific, Waltham, MA, USA).

### 4.18. Statistical Analysis

Data were presented as mean ± SD. Differences between two groups and multiple groups were assessed by paired/unpaired *t*-test and one-way ANOVA, respectively. Correlation between ENO1 and ApoC3 expression was identified using Pearson’s correlation analysis. Here, *p* < 0.05 was assumed as a statistically significant difference. GraphPad Prism version 7.0 for Windows (GraphPad Software, San Diego, CA, USA) was used for statistical analyses.

## Figures and Tables

**Table 1:** Proteins interacting with ENO1 in postoperative lymphatic drainage (PLD) from patients with oral squamous cell carcinoma (OSCC) (*n* = 4).

Accession	Gene	Identified Proteins	Coverage	Peptides
P02042	HBD	Hemoglobin subunit delta	70.07	37
A0A0C4DH69	IGKV1-9	Immunoglobulin kappa variable 1-9	48.72	5
A0A0C4DH38	IGHV5-51	Immunoglobulin heavy variable 5-51	45.30	6
P02656	APOC3	Apolipoprotein C-III	44.44	3
P01611	IGKV1D-12	Immunoglobulin kappa variable 1D-12	43.59	7
P09382	LGALS1	Galectin-1	42.21	4
A0A087WSY6	IGKV3D-15	Immunoglobulin kappa variable 3D-15	40.00	8
P00915	CA1	Carbonic anhydrase 1	35.62	7
P61626	LYZ	Lysozyme C	35.13	3
P06312	IGKV4-1	Immunoglobulin kappa variable 4-1	34.70	7

## Data Availability

The datasets generated during and/or analyzed during this study are available from the corresponding author upon reasonable request.

## Supplementary Materials

The supporting information can be downloaded at: https://www.mdpi.com/article/10.3390/ijms232112777/s1.
