# Supplementary material for: ENO1 Binds to ApoC3 and Impairs the Proliferation of T Cells via IL-8/STAT3 Pathway in OSCC
Source: Int J Mol Sci. 2022 Oct 24;23(21):12777. doi: 10.3390/ijms232112777 (PMC9654972; doi:10.3390/ijms232112777)
Supplement: Supplementary file 1 [file ijms-23-12777-s001.zip › ijms-1983672-supplementary.pdf]

ENO1 Binds to ApoC3 and Impairs the Proliferation of T Cells via IL-8/STAT3 Pathway in OSCC

Jing Wang[1, #], Qi-Wen Man[1, 2, #], Nian-Nian Zhong[1], Han-Qi Wang[1], Chen-Xi Zhang[1], Su-Ran Li[1], Lin-Lin Bu[1, 2, *], Bing Liu[1, 2, *]

[1] The State Key Laboratory Breeding Base of Basic Science of Stomatology (Hubei-MOST) & Key Laboratory of Oral Biomedicine Ministry of Education, School & Hospital of Stomatology, Wuhan University, Wuhan, China.

[2] Department of Oral and Maxillofacial Head Neck Oncology, School & Hospital of Stomatology, Wuhan University, Wuhan, China.

[#] Equal contributors

[*]Corresponding authors:

Lin-Lin Bu, DDS, MD, Associate Professor, Email: lin-lin.bu@whu.edu.cn;

Bing Liu, DDS, MD, Professor, Email: liubing9909@whu.edu.cn

Tel./Fax: +86 87686215

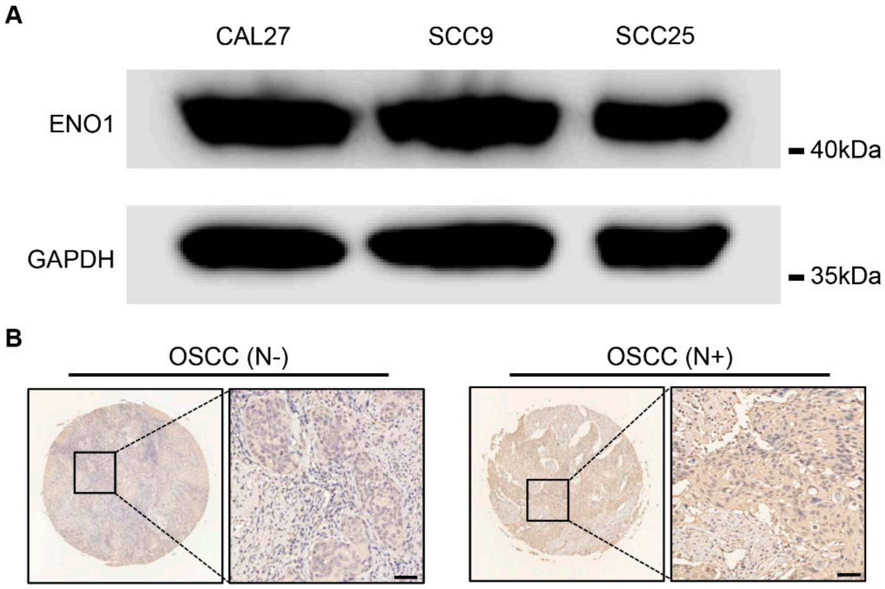

Figure S1. Overexpression and clinicopathological significance of ENO1 in OSCC. **(A)** Western blot analysis was assessed to examine ENO1 expression in OSCC cell lines. **(B)** Representative IHC staining of ENO1 in non-metastatic and metastatic OSCC tissues. Scale bar, 50 μm.

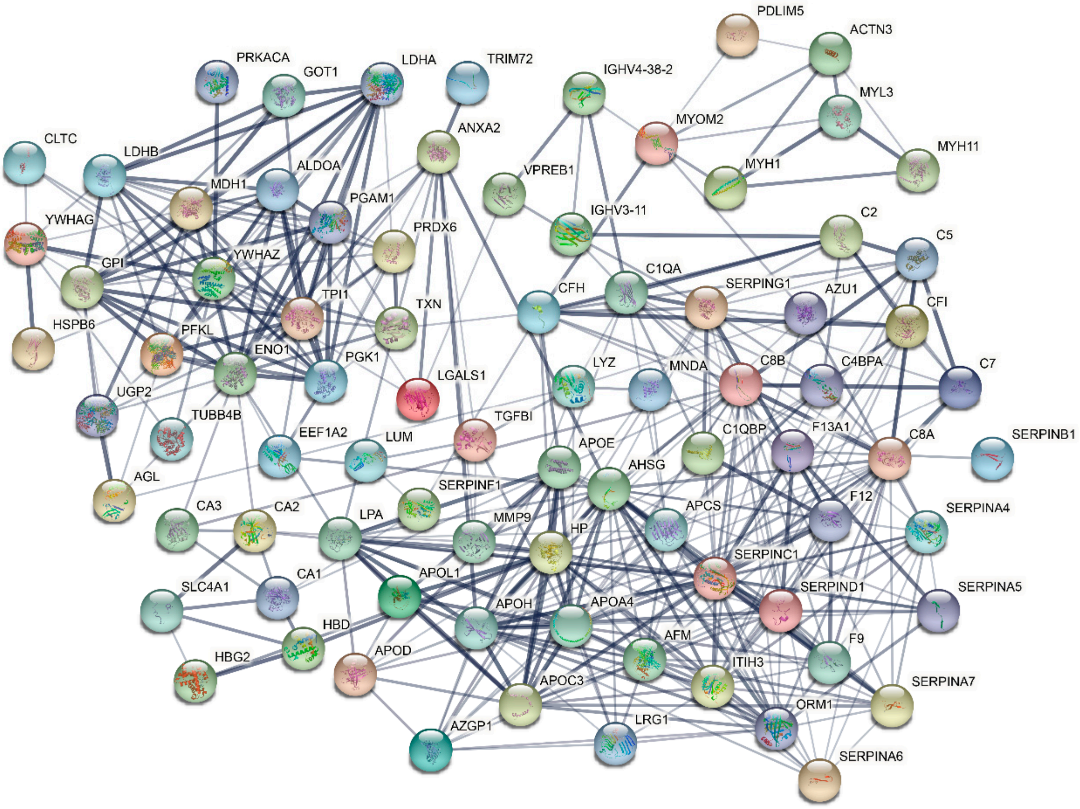

Figure S2. Protein-protein interaction (PPI) network of ENO1 interacting proteins in postoperative lymphatic drainage.

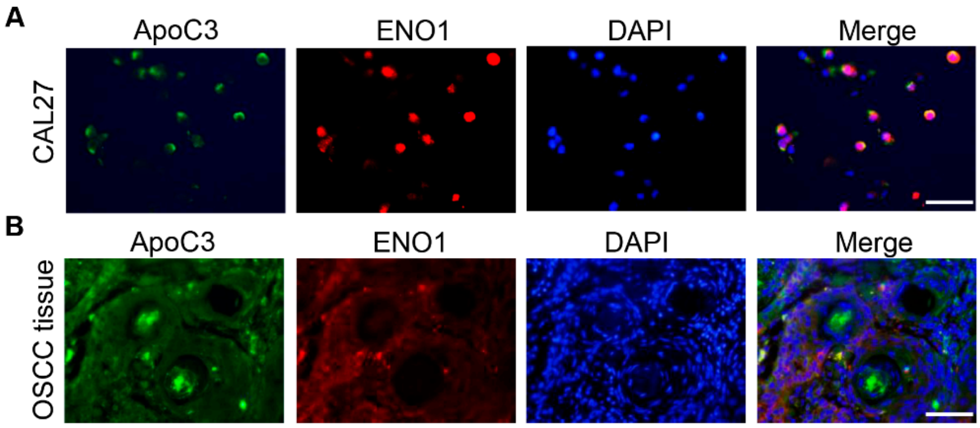

Figure S3. ENO1 bound to ApoC3 in OSCC. **(A)** Double-labelling immunofluorescence staining for ApoC3 (green) and ENO1 (red) in CAL27 cells. **(B)** Double-labelling immunofluorescence staining for ApoC3 (green) and ENO1 (red) in OSCC tissue. Scale bar, 50 μm.

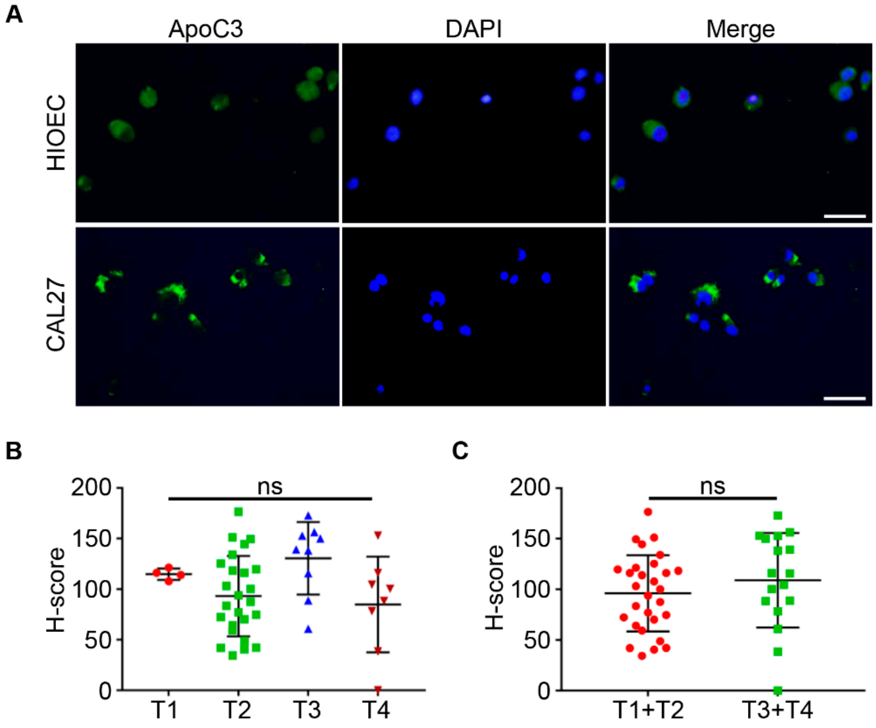

Figure S4. Overexpression and clinicopathological significance of ApoC3 in OSCC. **(A)** Immunofluorescence for ApoC3 in HIOEC and CAL27 cells. **(B)** Quantitative analysis of the ApoC3 expression of in OSCC classified by tumor sizes (T1, T2, T3, and T4). **(C)** Quantitative analysis of the ApoC3 expression of in OSCC classified by tumor sizes (T1+T2, T3+T4). ns is not significant.

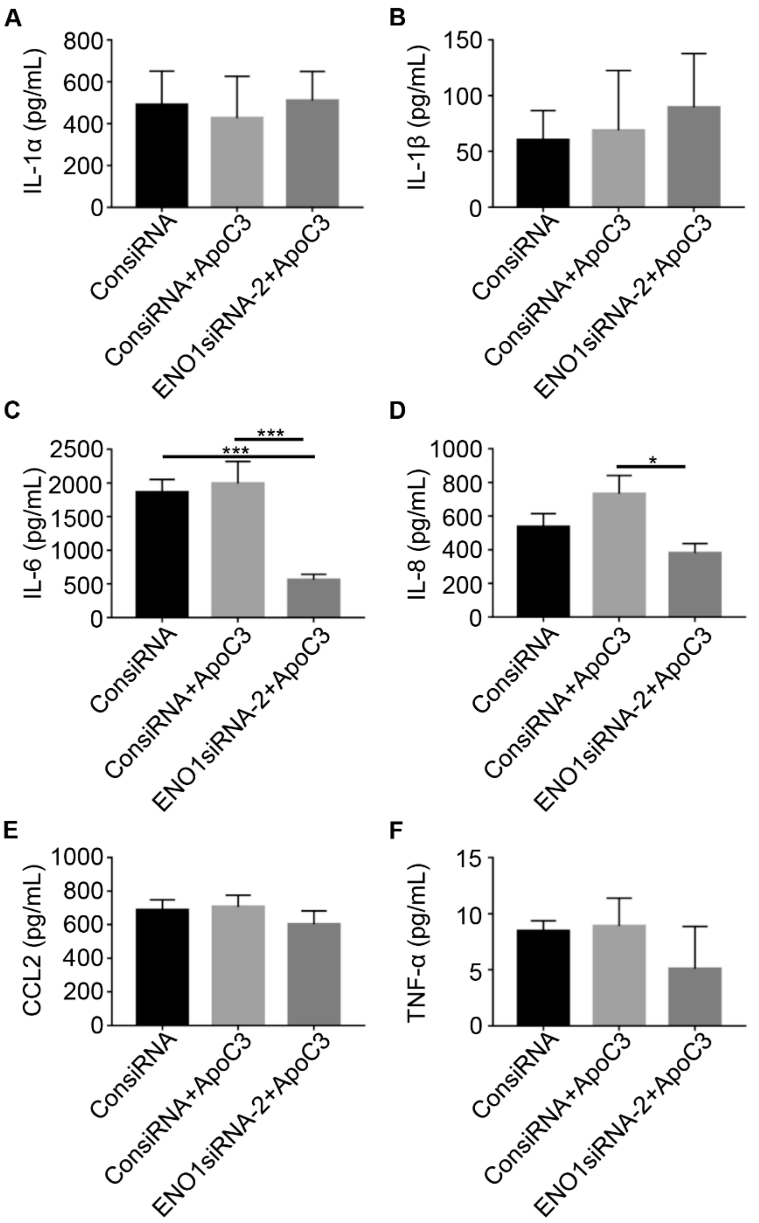

Figure S5. Inflammatory cytokine levels in cultured CAL27 supernatants. **(A-F)** showed the expression levels of IL-1α, IL-1β, IL-6, IL-8, CCL2, TNF-α, respectively. * $p < 0.05$; *** $p < 0.001$.

Table S1. Proteins (n=100) interacting with ENO1 postoperative lymphatic drainage (PLD) from patients with oral squamous cell carcinoma (OSCC) (n=4).

Accession	Gene	Protein Name	Coverage	Peptides
P02042	HBD	Hemoglobin subunit delta	70.07	37
A0A0C4DH69	IGKV1-9	Immunoglobulin kappa variable 1-9	48.72	5
A0A0C4DH38	IGHV5-51	Immunoglobulin heavy variable 5-51	45.3	6
P02656	APOC3	Apolipoprotein C-III	44.44	3
P01611	IGKV1D-12	Immunoglobulin kappa variable 1D-12	43.59	7
P09382	LGALS1	Galectin-1	42.22	4
A0A087WSY6	IGKV3D-15	Immunoglobulin kappa variable 3D-15	40	8
P00915	CA1	Carbonic anhydrase 1	35.63	7
P61626	LYZ	Lysozyme C	35.14	3
P06312	IGKV4-1	Immunoglobulin kappa variable 4-1	34.71	5
P04433	IGKV3-11	Immunoglobulin kappa variable 3-11	33.91	20
P01008	SERPINC1	Antithrombin-III	31.9	12
P01602	IGKV1-5	Immunoglobulin kappa variable 1-5	30.77	7
P04430	IGKV1-16	Immunoglobulin kappa variable 1-16	30.77	7
P30041	PRDX6	Peroxiredoxin-6	29.46	5
P01594	IGKV1-33	Immunoglobulin kappa variable 1-33	29.06	6
P04075	ALDOA	Fructose-bisphosphate aldolase A	27.2	10
A0A0A0MT36	IGKV6D-21	Immunoglobulin kappa variable 6D-21	24.56	2
P06727	APOA4	Apolipoprotein A-IV	23.74	8
P02763	ORM1	Alpha-1-acid glycoprotein 1	23.38	5
P02743	APCS	Serum amyloid P-component	21.97	5
P02765	AHSG	Alpha-2-HS-glycoprotein	21.25	7

P01861	IGHG4	Immunoglobulin heavy constant gamma 4	18.65	11
P25311	AZGP1	Zinc-alpha-2-glycoprotein	18.46	4
P00918	CA2	Carbonic anhydrase 2	18.08	3
P69892	HBG2	Hemoglobin subunit gamma-2	15.65	6
O14558	HSPB6	Heat shock protein beta-6	15	2
Q9BTM1	H2AJ	Histone H2A.J	14.73	2
P02745	C1QA	Complement C1q subcomponent subunit A	14.69	3
A0A075B6H9	IGLV4-69	Immunoglobulin lambda variable 4-69	14.29	1
P07451	CA3	Carbonic anhydrase 3	14.23	5
A0A087WSX0	IGLV5-45	Immunoglobulin lambda variable 5-45	13.82	3
P0DP08	IGHV4-38-2	Immunoglobulin heavy variable 4-38-2	13.68	5
P80748	IGLV3-21	Immunoglobulin lambda variable 3-21	13.68	1
P08603	CFH	Complement factor H	12.75	12
P10599	TXN	Thioredoxin	12.38	1
A0A0B4J1V2	IGHV2-26	Immunoglobulin heavy variable 2-26	11.76	2
P60174	TPI1	Triosephosphate isomerase	11.65	2
P07195	LDHB	L-lactate dehydrogenase B chain	10.78	4
P00338	LDHA	L-lactate dehydrogenase A chain	10.54	5
P07357	C8A	Complement component C8 alpha chain	10.27	3
P05155	SERPING1	Plasma protease C1 inhibitor	10.2	5
P14780	MMP9	Matrix metalloproteinase-9	10.04	6
P35080	PFN2	Profilin-2	10	1

P40925	MDH1	Malate dehydrogenase, cytoplasmic	9.88	2
P18669	PGAM1	Phosphoglycerate mutase 1	9.84	2
P63104	YWHAZ	14-3-3 protein zeta/delta	9.8	2
P61981	YWHAG	14-3-3 protein gamma	9.72	2
P12882	MYH1	Myosin-1	9.7	18
P08185	SERPINA6	Corticosteroid-binding globulin	9.63	3
P02749	APOH	Beta-2-glycoprotein 1	9.57	6
P17174	GOT1	Aspartate aminotransferase, cytoplasmic	8.47	2
Q05639	EEF1A2	Elongation factor 1-alpha 2	8.42	2
P00558	PGK1	Phosphoglycerate kinase 1	8.15	2
P68371	TUBB4B	Tubulin beta-4B chain	8.09	3
P05090	APOD	Apolipoprotein D	7.94	1
P51884	LUM	Lumican OS=Homo sapiens	7.69	2
P08590	MYL3	Myosin light chain 3	7.69	1
P05156	CFI	Complement factor I	6.86	3
P43652	AFM	Afamin OS=Homo sapiens	6.68	3
Q08043	ACTN3	Alpha-actinin-3	6.55	6
P00488	F13A1	Coagulation factor XIII A chain	6.42	3
P36955	SERPINF1	Pigment epithelium-derived factor	5.98	2
P07358	C8B	Complement component C8 beta chain	5.92	3
P05154	SERPINA5	Plasma serine protease inhibitor	5.91	2
Q16851	UGP2	UTP--glucose-1-phosphate uridylyltransferase	5.91	2
Q92947	GCDH	Glutaryl-CoA dehydrogenase, mitochondrial	5.48	1

P47755	CAPZA2	F-actin-capping protein subunit alpha-2	5.25	1
Q6ZMU5	TRIM72	Tripartite motif-containing protein 72	5.24	2
P20160	AZU1	Azurocidin	5.18	2
P06744	GPI	Glucose-6-phosphate isomerase	5.018	2
P05546	SERPIND1	Heparin cofactor 2	5.01	3
P10643	C7	Complement component C7	4.98	3
Q07021	C1QBP	Complement component 1 Q subcomponent-binding protein, mitochondrial	4.96	1
P02750	LRG1	Leucine-rich alpha-2-glycoprotein	4.61	1
P02649	APOE	Apolipoprotein E	4.42	1
Q06033	ITIH3	Inter-alpha-trypsin inhibitor heavy chain H3	4.38	4
Q96HC4	PDLIM5	PDZ and LIM domain protein 5	4.36	3
P41218	MNDA	Myeloid cell nuclear differentiation antigen	4.18	1
O14791	APOL1	Apolipoprotein L1	3.77	1
P30740	SERPINB1	Leukocyte elastase inhibitor	3.69	1
Q15582	TGFBI	Transforming growth factor-beta-induced protein ig-h3	3.66	2
P0DOX4	--	Immunoglobulin epsilon heavy chain	3.47	2
P17612	PRKACA	cAMP-dependent protein kinase catalytic subunit alpha	3.42	1
P07355	ANXA2	Annexin A2	3.25	1
P54296	MYOM2	Myomesin-2	3	3
P00748	F12	Coagulation factor XII	2.93	1
P01031	C5	Complement C5	2.75	3
P05543	SERPINA7	Thyroxine-binding globulin	2.41	1

P02730	SLC4A1	Band 3 anion transport protein	2.31	2
P00738	HP	Haptoglobin	2.22	1
P17858	PFKL	ATP-dependent 6-phosphofructokinase, liver type	2.18	1
P29622	SERPINA4	Kallistatin OS=Homo sapiens	2.11	1
P04003	C4BPA	C4b-binding protein alpha chain	2.01	1
P00740	F9	Coagulation factor IX	1.95	1
P06681	C2	Complement C2	1.6	1
P08519	LPA	Apolipoprotein(a)	1.21	6
P35573	AGL	Glycogen debranching enzyme	0.91	1
P35749	MYH11	Myosin-11	0.81	1
Q00610	CLTC	Clathrin heavy chain 1	0.54	1

Table S2. The complete list of the enriched KEGG pathways.

No.	Term	Pathway ID	Protein number	Protein
1	Metabolic pathways	hsa01100	17	P00338; P35573; P40925; P04075; P60174; P07451; P17858; Q16851; P00918; P18669; P30041; P00558; P06744; P07195; P17174; Q92947; P00915
2	Complement and coagulation cascades	hsa04610	16	P07358; P00748; P07357; P08603; P00488; P05546; P01031; P05154; P05155; P00740; P04003; P01008; P05156; P02745; P10643; P06681
3	Coronavirus disease - COVID-19	hsa05171	10	P0DP08; A0A0C4DH38; P07358; P07357; P02745; P10643; P00488; P06681; A0A0B4J1V2; P01031
4	Systemic lupus erythematosus	hsa05322	10	P0DP08; A0A0C4DH38; P07358; Q9BTM1; P07357; P02745; P10643; P06681; A0A0B4J1V2; P01031
5	Carbon metabolism	hsa01200	8	P17174; P40925; P04075; P60174; P17858; P18669; P00558; P06744
6	Glycolysis / Gluconeogenesis	hsa00010	8	P00338; P04075; P60174; P17858; P18669; P00558; P07195; P06744
7	Staphylococcus aureus infection	hsa05150	8	P0DP08; A0A0C4DH38; P05156; P02745; P08603; P06681; P01031; A0A0B4J1V2
8	Prion disease - Homo sapiens	hsa05020	7	P68371; P07357; P02745; P10643; P07358; P17612; P01031
9	Amoebiasis - Homo sapiens	hsa05146	6	P07357; P0DP08; A0A0C4DH38; P07358; A0A0B4J1V2; P17612
10	Biosynthesis of amino acids	hsa01230	6	P17174; P04075; P18669; P60174; P17858; P00558

11	Cholesterol metabolism	hsa04979	5	P06727; P02656; P02649; P02749; P08519
12	Dilated cardiomyopathy	hsa05414	5	P08590; P0DP08; A0A0C4DH38; A0A0B4J1V2; P17612
13	Glucagon signaling pathway	hsa04922	5	P00338; P18669; P17858; P17612; P07195
14	HIF-1 signaling pathway	hsa04066	5	P00338; P04075; P17858; P00558; P07195
15	Pathogenic Escherichia coli infection	hsa05130	5	P68371; P0DP08; A0A0C4DH38; P35749; A0A0B4J1V2
16	Pertussis	hsa05133	5	P02745; P04003; P06681; P01031; P05155
17	PI3K-Akt signaling pathway	hsa04151	5	P61981; P0DP08; A0A0C4DH38; A0A0B4J1V2; P63104
18	African trypanosomiasis	hsa05143	4	P0DP08; A0A0C4DH38; O14791; A0A0B4J1V2
19	Calcium signaling pathway	hsa04020	4	P0DP08; A0A0C4DH38; A0A0B4J1V2; P17612
20	Central carbon metabolism in cancer	hsa05230	4	P00338; P18669; P17858; P07195
21	Cysteine and methionine metabolism	hsa00270	4	P00338; P17174; P40925; P07195
22	Leishmaniasis	hsa05140	4	Q05639; P0DP08; A0A0C4DH38; A0A0B4J1V2
23	Phagosome	hsa04145	4	P68371; P0DP08; A0A0C4DH38; A0A0B4J1V2
24	Salmonella infection	hsa05132	4	P68371; P35080; P10599; P07355
25	Transcriptional misregulation in cancer	hsa05202	4	P14780; P0DP08; A0A0C4DH38; A0A0B4J1V2
26	Allograft rejection	hsa05330	3	P0DP08; A0A0C4DH38; A0A0B4J1V2
27	Asthma	hsa05310	3	P0DP08; A0A0C4DH38; A0A0B4J1V2
28	Autoimmune thyroid disease	hsa05320	3	P0DP08; A0A0C4DH38; A0A0B4J1V2

29	B cell receptor signaling pathway	hsa04662	3	P0DP08; A0A0C4DH38; A0A0B4J1V2
30	Epstein-Barr virus infection	hsa05169	3	P0DP08; A0A0C4DH38; A0A0B4J1V2
31	Fc epsilon RI signaling pathway	hsa04664	3	P0DP08; A0A0C4DH38; A0A0B4J1V2
32	Fc gamma R-mediated phagocytosis	hsa04666	3	P0DP08; A0A0C4DH38; A0A0B4J1V2
33	Fructose and mannose metabolism	hsa00051	3	P04075; P60174; P17858
34	Hematopoietic cell lineage	hsa04640	3	P0DP08; A0A0C4DH38; A0A0B4J1V2
35	Intestinal immune network for IgA production	hsa04672	3	P0DP08; A0A0C4DH38; A0A0B4J1V2
36	Natural killer cell mediated cytotoxicity	hsa04650	3	P0DP08; A0A0C4DH38; A0A0B4J1V2
37	NF-kappa B signaling pathway	hsa04064	3	P0DP08; A0A0C4DH38; A0A0B4J1V2
38	Nitrogen metabolism	hsa00910	3	P07451; P00918; P00915
39	Oocyte meiosis	hsa04114	3	P61981; P17612; P63104
40	Parkinson disease	hsa05012	3	P68371; P10599; P17612
41	Pentose phosphate pathway	hsa00030	3	P04075; P17858; P06744
42	Phospholipase D signaling pathway	hsa04072	3	P0DP08; A0A0C4DH38; A0A0B4J1V2
43	Primary immunodeficiency	hsa05340	3	P0DP08; A0A0C4DH38; A0A0B4J1V2
44	Proteoglycans in cancer	hsa05205	3	P51884; P14780; P17612
45	Pyruvate metabolism	hsa00620	3	P00338; P40925; P07195
46	Rheumatoid arthritis	hsa05323	3	P0DP08; A0A0C4DH38; A0A0B4J1V2
47	Starch and sucrose metabolism	hsa00500	3	P35573; Q16851; P06744
48	Tuberculosis	hsa05152	3	P0DP08; A0A0C4DH38; A0A0B4J1V2
49	Viral carcinogenesis	hsa05203	3	P61981; P17612; P63104
50	Viral myocarditis	hsa05416	3	P0DP08; A0A0C4DH38; A0A0B4J1V2
51	Yersinia infection	hsa05135	3	P0DP08; A0A0C4DH38; A0A0B4J1V2

52	Adrenergic signaling in cardiomyocytes	hsa04261	2	P08590; P17612
53	Alcoholism	hsa05034	2	Q9BTM1; P17612
54	Alzheimer disease	hsa05010	2	P68371; P02649
55	Amino sugar and nucleotide sugar metabolism	hsa00520	2	Q16851; P06744
56	Amyotrophic lateral sclerosis	hsa05014	2	P68371; P35080
57	Apelin signaling pathway	hsa04371	2	P08590; P17612
58	Bile secretion	hsa04976	2	P17612; P00918
59	Cell cycle	hsa04110	2	P61981; P63104
60	Collecting duct acid secretion	hsa04966	2	P02730; P00918
61	Endocrine and other factor-regulated calcium reabsorption	hsa04961	2	P17612; Q00610
62	Endocrine resistance	hsa01522	2	P14780; P17612
63	Endocytosis	hsa04144	2	P47755; Q00610
64	Estrogen signaling pathway	hsa04915	2	P14780; P17612
65	Fluid shear stress and atherosclerosis	hsa05418	2	P14780; P10599
66	Galactose metabolism	hsa00052	2	P17858; Q16851
67	Gap junction	hsa04540	2	P68371; P17612
68	Gastric acid secretion	hsa04971	2	P17612; P00918
69	Hepatitis B	hsa05161	2	P14780; P63104
70	Hepatitis C	hsa05160	2	P61981; P63104
71	Hippo signaling pathway	hsa04390	2	P61981; P63104
72	Huntington disease	hsa05016	2	P68371; Q00610
73	Pathways in cancer	hsa05200	2	P14780; P17612
74	Propanoate metabolism	hsa00640	2	P00338; P07195
75	Proximal tubule bicarbonate reclamation	hsa04964	2	P40925; P00918
76	Regulation of actin cytoskeleton	hsa04810	2	P35080; P35749
77	Relaxin signaling pathway	hsa04926	2	P14780; P17612
78	Salivary secretion	hsa04970	2	P17612; P61626

79	Thyroid hormone signaling pathway	hsa04919	2	P17858; P17612
80	Thyroid hormone synthesis	hsa04918	2	P17612; P05543
81	Tight junction	hsa04530	2	P35749; P17612
82	Vascular smooth muscle contraction	hsa04270	2	P35749; P17612
83	Wnt signaling pathway	hsa04310	2	P36955; P17612
84	2-Oxocarboxylic acid metabolism	hsa01210	1	P17174
85	Alanine, aspartate and glutamate metabolism	hsa00250	1	P17174
86	Aldosterone synthesis and secretion	hsa04925	1	P17612
87	Amphetamine addiction	hsa05031	1	P17612
88	AMPK signaling pathway	hsa04152	1	P17858
89	Arginine and proline metabolism	hsa00330	1	P17174
90	Arginine biosynthesis	hsa00220	1	P17174
91	Arrhythmogenic right ventricular cardiomyopathy	hsa05412	1	Q08043
92	Autophagy - animal	hsa04140	1	P17612
93	Bacterial invasion of epithelial cells	hsa05100	1	Q00610
94	Biosynthesis of cofactors	hsa01240	1	Q16851
95	Bladder cancer	hsa05219	1	P14780
96	cAMP signaling pathway	hsa04024	1	P17612
97	Cardiac muscle contraction	hsa04260	1	P08590
98	Chagas disease	hsa05142	1	P02745
99	Chemokine signaling pathway	hsa04062	1	P17612
100	Cholinergic synapse	hsa04725	1	P17612
101	Circadian entrainment	hsa04713	1	P17612
102	Citrate cycle (TCA cycle)	hsa00020	1	P40925
103	Cocaine addiction	hsa05030	1	P17612

104	Cortisol synthesis and secretion	hsa04927	1	P17612
105	Cushing syndrome	hsa04934	1	P17612
106	Dopaminergic synapse	hsa04728	1	P17612
107	Fat digestion and absorption	hsa04975	1	P06727
108	Fatty acid degradation	hsa00071	1	Q92947
109	GABAergic synapse	hsa04727	1	P17612
110	Glutamatergic synapse	hsa04724	1	P17612
111	Glutathione metabolism	hsa00480	1	P30041
112	Glycine, serine and threonine metabolism	hsa00260	1	P18669
113	Glyoxylate and dicarboxylate metabolism	hsa00630	1	P40925
114	GnRH signaling pathway	hsa04912	1	P17612
115	Growth hormone synthesis, secretion and action	hsa04935	1	P17612
116	Hedgehog signaling pathway	hsa04340	1	P17612
117	Herpes simplex virus 1 infection	hsa05168	1	P01031
118	Human cytomegalovirus infection	hsa05163	1	P17612
119	Human papillomavirus infection	hsa05165	1	P17612
120	Human T-cell leukemia virus 1 infection	hsa05166	1	P17612
121	Hypertrophic cardiomyopathy	hsa05410	1	P08590
122	IL-17 signaling pathway	hsa04657	1	P14780
123	Inflammatory mediator regulation of TRP channels	hsa04750	1	P17612

124	Inositol phosphate metabolism	hsa00562	1	P60174
125	Insulin secretion	hsa04911	1	P17612
126	Insulin signaling pathway	hsa04910	1	P17612
127	Legionellosis	hsa05134	1	Q05639
128	Leukocyte transendothelial migration	hsa04670	1	P14780
129	Longevity regulating pathway	hsa04211	1	P17612
130	Longevity regulating pathway - multiple species	hsa04213	1	P17612
131	Long-term potentiation	hsa04720	1	P17612
132	Lysine degradation	hsa00310	1	Q92947
133	Lysosome	hsa04142	1	Q00610
134	MAPK signaling pathway	hsa04010	1	P17612
135	Melanogenesis	hsa04916	1	P17612
136	MicroRNAs in cancer	hsa05206	1	P14780
137	Morphine addiction	hsa05032	1	P17612
138	Necroptosis	hsa04217	1	Q9BTM1
139	Neuroactive ligand-receptor interaction	hsa04080	1	P01031
140	NOD-like receptor signaling pathway	hsa04621	1	P10599
141	Olfactory transduction	hsa04740	1	P17612
142	Ovarian steroidogenesis	hsa04913	1	P17612
143	Oxytocin signaling pathway	hsa04921	1	P17612
144	Pancreatic secretion	hsa04972	1	P00918
145	Parathyroid hormone synthesis, secretion and action	hsa04928	1	P17612
146	Pathways of neurodegeneration - multiple diseases	hsa05022	1	P68371
147	Pentose and glucuronate interconversions	hsa00040	1	Q16851

148	Phenylalanine metabolism	hsa00360	1	P17174
149	Phenylalanine, tyrosine and tryptophan biosynthesis	hsa00400	1	P17174
150	Platelet activation	hsa04611	1	P17612
151	PPAR signaling pathway	hsa03320	1	P02656
152	Progesterone-mediated oocyte maturation	hsa04914	1	P17612
153	Prostate cancer	hsa05215	1	P14780
154	Rap1 signaling pathway	hsa04015	1	P35080
155	Ras signaling pathway	hsa04014	1	P17612
156	Regulation of lipolysis in adipocytes	hsa04923	1	P17612
157	Renin secretion	hsa04924	1	P17612
158	Retrograde endocannabinoid signaling	hsa04723	1	P17612
159	RNA degradation	hsa03018	1	P17858
160	RNA transport	hsa03013	1	Q05639
161	Serotonergic synapse	hsa04726	1	P17612
162	Shigellosis	hsa05131	1	P35080
163	Synaptic vesicle cycle	hsa04721	1	Q00610
164	Taste transduction	hsa04742	1	P17612
165	Thermogenesis	hsa04714	1	P17612
166	TNF signaling pathway	hsa04668	1	P14780
167	Tryptophan metabolism	hsa00380	1	Q92947
168	Tyrosine metabolism	hsa00350	1	P17174
169	Vasopressin-regulated water reabsorption	hsa04962	1	P17612
170	Vibrio cholerae infection	hsa05110	1	P17612
171	Vitamin digestion and absorption	hsa04977	1	P06727
